# Uncoupling human and climate drivers of late Holocene vegetation change in southern Brazil

**DOI:** 10.1038/s41598-018-24429-5

**Published:** 2018-05-17

**Authors:** Mark Robinson, Jonas Gregorio De Souza, S. Yoshi Maezumi, Macarena Cárdenas, Luiz Pessenda, Keith Prufer, Rafael Corteletti, Deisi Scunderlick, Francis Edward Mayle, Paulo De Blasis, José Iriarte

**Affiliations:** 1 Department of Archaeology, University of Exeter, 222 Laver Building, North Park Road, Exeter, Devon, EX4 4QE UK; 2 Centre for Past Climate Change and Department of Geography & Environmental Science, University of Reading, Reading, RG6 6DW UK; 3 Centre of Nuclear Energy in Agriculture, University of São Paulo, Piracicaba, 13416-000 Brazil; 4 Department of Anthropology, University of New Mexico, MCS01-1040, Albuquerque, NM 87106 USA; 5 Instituto de Ciências Humanas, Departamento de Antropologia e Arqueologia, Universidade Federal de Pelotas, Rua Cel. Alberto Rosa, 154, Pelotas, RS Brazil; 6 Universidade do Sul de Santa Caterina, Av. José Acácio Moreira 787, Bairro Dehon, 88.704-900 - Tubarão, Santa Catarina, Brazil; 7 Laboratório de Arqueologia Regional, Museu de Arqueologia e Etnologia, Universidade de São Paulo, São Paulo, Brazil

## Abstract

In the highlands of southern Brazil an anthropogenitcally driven expansion of forest occurred at the expense of grasslands between 1410 and 900 cal BP, coincident with a period of demographic and cultural change in the region. Previous studies have debated the relative contributions of increasing wetter and warmer climate conditions and human landscape modifications to forest expansion, but generally lacked high resoltiuon proxies to measure these effects, or have relied on single proxies to reconstruct both climate and vegetation. Here, we develop and test a model of natural ecosystem distribution against vegetation histories, paleoclimate proxies, and the archaeological record to distinguish human from temperature and precipitation impacts on the distribution and expansion of *Araucaria* forests during the late Holocene. Carbon isotopes from soil profiles confirm that in spite of climatic fluctuations, vegetation was stable and forests were spatially limited to south-facing slopes in the absence of human inputs. In contrast, forest management strategies for the past 1400 years expanded this economically important forest beyond its natural geographic boundaries in areas of dense pre-Columbian occupation, suggesting that landscape modifications were linked to demographic changes, the effects of which are still visible today.

## Introduction

In the face of global climate change and intensifying population pressure, understanding the drivers of vegetation change is critical for developing appropriate conservation practices to secure global biodiversity. A pressing issue is the legacy of past human activity on landscapes. Traditional research methods have often been unable to uncouple the causal relationships among humans, climate, and vegetation, resulting in ongoing unresolved debates, which can result in ineffectual policy and can unnecessarily negatively impact local economies.

An area of particular significance is the southern Brazilian highlands, which is part of the Atlantic Forest Biome. Recognised as a global diversity hotspot, the area represents an important region for conserving global diversity due to its floristic importance and modern conservation challenges^1^. The keystone species of the forest is the iconic *Araucaria* tree. The tree is a valuable source of timber, fuel, and resin, and the seeds are an abundant and reliable food source that has been a critical component of indigenous economies. Today, of the 19 extant species, five are classified as endangered and two, including the Brazilian *Araucaria angustifolia*, are critically endangered.

Between 1410 and 900 cal BP, forest expansion in the southern Brazilian highlands coincided with the growth of indigenous southern proto Jê populations and increasingly complex cultural institutions. The principal driver of this dynamic period have been debated^2–6^. Did wetter climate drive forest expansion, providing a greater resource base that enabled community development? Or, did growing populations result in widespread management of the landscape to increase economically important biotic resources? Until now, no research group has been able to integrate and analyse the relevant datasets in a way to satisfactorily answer this major question.

**Figure 1:**
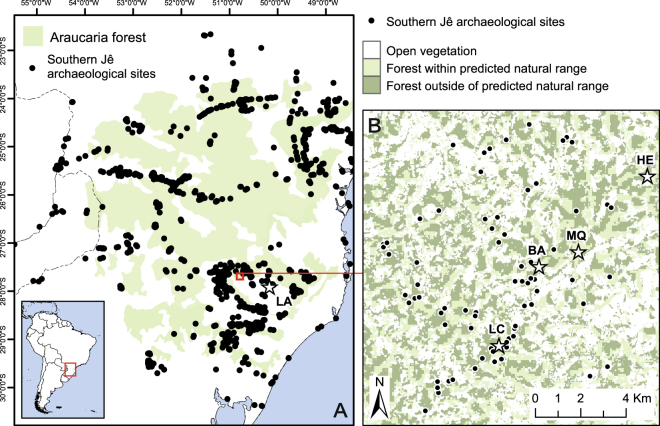
(**A**) Distribution of Southern Jê archaeological sites and the LA soil profile in relation to the extent of the *Araucaria* forests in southern Brazil. (**B**) Archaeological pilot area of Campo Belo do Sul, with archaeological sites and soil profiles in relation to predicted and actual distribution of *Araucaria* forest (see text). Soil profiles: LA = Lages; LC = Luís Carlos; BA = Baggio; MQ = Mato Queimado; HE = Heraldo.

In this study we develop and test an interdisciplinary methodology to distinguish human impacts from climate-induced vegetation changes in the southern Brazilian highlands (Fig. 1) during a period of abrupt cultural and environmental change. We compare original ecological and archaeological data to existing paleoclimate records from the region and provide a paleoecological dataset from Campo Belo do Sul, Santa Catarina State. Isotopic profiles of soil test pits are used to assess localised vegetation histories against a predictive model of natural forest distribution and a robust archaeological dataset^7–10^. Results challenge a dominant hypothesis of climatically driven Late Holocene *Araucaria* forest expansion, questioning the use of pollen datasets as a proxy to reconstruct both climate and vegetation history, and suggesting that regardless of climatic variability, vegetation was stable in areas of low human activity but forest expansion occurred at the expense of grasslands in areas of high archaeological activity. As such, regional vegetation dynamics and species distribution must be understood in relation to anthropogenic inputs.

## Environmental and Cultural Context

The Atlantic Forest Biome of southern Brazil is characterised by high species diversity and endemism across several ecoregions that stretch along the Brazilian coast from 18° to 30° S, and inland across the highlands into Paraguay and Argentina. An escarpment rises ~1000 m from the Atlantic coastal plain, abruptly marking the start of the highlands. The highlands slowly decrease in elevation to the Paraná and Uruguay River floodplains in the west, forming a dissected landscape of hills, ridges, plateaus, and valleys. The highland climate is humid mesothermic, with mean annual temperatures between 15–20 °C and mean annual rainfall of 1500–2000 mm. Modern vegetation is dominated by a mosaic of high altitude *campos* (grasslands) and Mixed Ombrophilous Forest, also known as *Araucaria* forest. In *Araucaria* forest, *Araucaria angustifolia* dominates the upper canopy and represents more than 40% of all individual trees. Several smaller-stature trees are also abundant: *Podocarpus lambertii, Ilex paraguayensis, Drymis brasiliensis, Symplocos uniflora*, and *Mimosa scabrella*^11,12^.

The distinctive *A. angustifolia* (commonly known as Paraná Pine, Brazilian pine, *pinheiro*), grows above 600 masl, becoming more dominant above 800 m. The species occurs between 24–30° S, in regions with annual rainfall >1400 mm and an average temperature range of 11.5 °C to 22 °C^2,13,14^. *Araucaria* has been characterised as a pioneer species and can act as a nurse plant for colonization under its canopy^14,15^. Gravity is the primary mechanism for seed dispersal; the heavy seeds requiring an agent for longer distance dispersal^3,6^. *Araucaria* is an important component of indigenous diets, producing large cones containing up to 200 large seeds that are rich in starch, dietary fibre, Mg, and Cu^16^. Culturally, the species is notable beyond its subsistence value, and is central to indigenous southern proto-Jê religion and social institutions^10,17–19^. Distribution of southern proto-Jê culture across the highlands is directly tied to the distribution of *Araucaria* forest, creating a legacy of cultural and ecological dynamics.

*Araucaria* produces firewood of exceptional quality, and is highly desired in construction due to its long straight trunk, durability and straight grain. Logging has been the primary cause of a 97% reduction of the *A. angustifolia* since the beginning of the 20^th^ century^20^, resulting in the species being assessed as Critically Endangered under the A2 criterion for the IUCN Red List. In addition to this decimation of the forest from modern logging, reports from the late 1800s describe trees with diameters of over 2 m, reaching 42 m in height^13^. These giants are no longer present. A modern study across 25 plots in Rio Grande do Sul recorded a mean height of just 17.7 m and a mean diameter at breast height of 0.38 m^21^.

**Figure 2:**
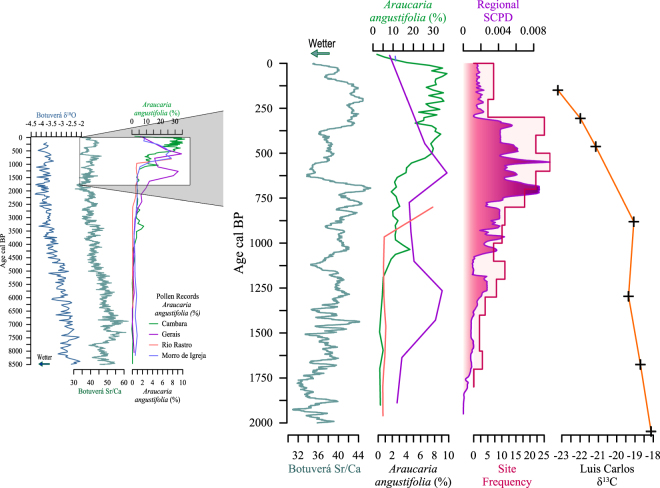
Synthesis of paleoclimate, regional pollen, regional archaeology data and paleoecological data at from the Luis Carlos in Campo Belo do Sul at 8.5ky and 2ky resolution. Luis Carlos δ^13^C isotopic profile of soil organic matter is plotted as an age-depth model based on three AMS dates. The 8.5ky record shows increasing precipitation from Sr/Ca and δ^18^O data in the Botuverá speleothem, without a corresponding increase in regional *Araucaria* vegetation. The 2ky record shows the increase in SCPD and vegetation transition beginning prior to the wetter period between ca. 600 and 350 BP. Pollen data are charted as a percentage of total pollen counts.

Pollen cores and isotopic soil profiles reveal an expansion of *Araucaria* forest at the expense of *campos* grassland vegetation throughout the southern Brazilian highlands in the Late Holocene (Fig. 2). An initial increase in *Araucaria* forest is recorded between 4480–3200 cal BP. In Santa Catarina state, the Serra da Boa Vista and Morro da Igreja wetland pollen cores, record an increase in taxa associated with *Araucaria* forest after 3760 cal BP^22^ and 2430 cal BP, respectively. In Rio Grande do Sul State, the Cambará do Sul pollen core records an initial forest expansion from 4320 cal BP^23^. During this time, forest expansion likely occurred along streams to form a network of gallery forest^2^. An increase in macroscopic charcoal around this time is likely related to the clearance of land of crop cultivation, although permanent settlements and earthwork architecture were still absent. These initial increases were subsequently halted until the onset of a rapid and extensive expansion of the forest, beginning between ca. 1410 and 900 cal BP^2,24–26^.

Pollen records across the region show a dramatic increase in *Araucaria* forest and replacement of grasslands after ca. 1410 cal BP. Forest patches expanded beyond the valley habitats into the adjacent highlands, forming a mosaic of forest and grasslands. This forest expansion began ca. 1440 cal BP in Serra dos Campos Gerais, Paraná State, followed by Cambará do Sul at ca. 1100 cal BP, São Francisco de Paula in Rio Grande do Sul ca. 990 cal BP, and Morro da Igreja and Serra do Rio Rastro in Santa Catarina ca. 900 cal BP^3,4^. Forest expansion has also been recorded in isotopic soil profiles from Rio Grande do Sul, starting after 1500–1300 cal BP, providing localised evidence of an increased contribution of C_3_ plants into soil formation on previously C_4_ grasslands^25^.

Stratigraphic changes in floristic composition have been interpreted as an adaptive response to a transition to a wetter, warmer, and less seasonal climate^2,4^. These later climatic conditions favour the adaptations of forest taxa over the declining dry adapted *campos* inventory. In this way, pollen based vegetation reconstructions were used by to infer past climatic change. However, exploring the relationship between forest expansion and climate change is highly problematic when the same proxy has been used to reconstruct both.

A speleothem from Botuverá cave in southern Brazil^27^ provides an isotopic paleoclimate rainfall proxy (Fig. 2). No significant change in precipitation is recorded during the period of forest expansion between 1000 and 700 cal BP. Both Sr/Ca and δ^18^O ratios indicate a gradual increase in precipitation from ca. 7000 to 4000 cal BP, at which point precipitation levelled out, with minor fluctuations for the next 2000 years^27^. Southern Brazil experienced its wettest period in the last 8000 years around 2000–1750 cal BP; however, this was followed by a slight drying trend during the onset of major forest expansion (ca. 1410 and 900 cal BP).

During this time of forest expansion, regional settlements and populations of southern proto-Jê speakers increased in size and distribution, accompanied by the development of public architectural forms^7–9,18,28^. As a generalised indicator of human activity across the whole highlands, Sum of the Calibrated Probability Distributions (SCPDs) suggest changes in population dynamics based on 245 AMS ^14^C radiocarbon dates (Fig. 2; Supplementary Tables S1 and S2). An initial population expansion around ca. 1400 cal BP was followed by a second wave at ca. 1100 cal BP that peaked after 800 cal BP. Population numbers and cultural activity declined after ca. 400 cal BP, associated with the impacts of European settlers. The earliest archaeologically identified permanent settlement of the Campo Belo do Sul region began at ca. 1020 cal BP. Domestic settlement increased across the area, reaching a peak ca. 650-400 cal BP. As in other areas, activity drastically declined after ca. 300 cal BP (Supplementary Table S1). Regional population numbers did not begin to recover until the 19^th^ century, when loggers began exploiting the *Araucaria* forests for timber.

## Modelling vegetation

Maximum entropy models can predict species distribution based on the geographic characteristics from the location of modern specimens^29,30^. We apply a similar concept, except we do not consider all modern examples to inhabit their natural environment; instead we derive the geographic characteristics from areas with minimal human disturbance that more accurately reflect natural distributions. *Araucaria* is present on the landscape in distinct patterns^31^. In particular, in regions of low past human impact, forests are present only on south-facing slopes, whereas north-facing slopes and plateaus are characterised by grassland vegetation. This pattern is likely the result of differential evapotranspiration caused by more direct sunlight on the north-facing slopes and increased wind shear from the prevailing north-easterly winds, which constrain the success of tree seeds. The influence of aspect on floristic composition and treeline elevation, due to its control over differential solar-radiation and moisture, has been described elsewhere, both in temperate latitudes and low latitude subtropics^32–34^.

A binomial logistic regression predicts forest distribution under natural conditions based on terrain variables. The data used to perform the regression come from a control region close to Lages, 60 km east of Campo Belo do Sul, representing the natural undisturbed conditions of *Araucaria* forest distribution with an absence of archaeological sites. All terrain variables were found to be significant in the prediction of forest distribution, and the standardised coefficients confirmed a negative correlation with hilltops and a positive correlation with south-facing slopes (Supplementary Table S3, see Methods). The predicted forest distribution is compared to modern vegetation cover using USGS satellite imagery from 1966 to avoid land cover impacts from the last 50 years of regional development. A significant positive correlation is found between the predicted and the actual forest distribution in 1966 (Pearson’s r = 0.232, p < 0.001).

When projected onto the archaeology-rich area of Campo Belo do Sul, there was no significant correlation between the model and actual forest distribution in 1966 (Pearson’s r = 0.002, p = 0.781). A separate regression run for Campo Belo do Sul with the same parameters resulted in lower standardised coefficients, implying less influence of the terrain variables in the distribution of the forest. Of particular interest is the positive, though weak, correlation with hilltops, contrary to the expected natural distribution (Supplementary Table S3).

**Figure 3:**
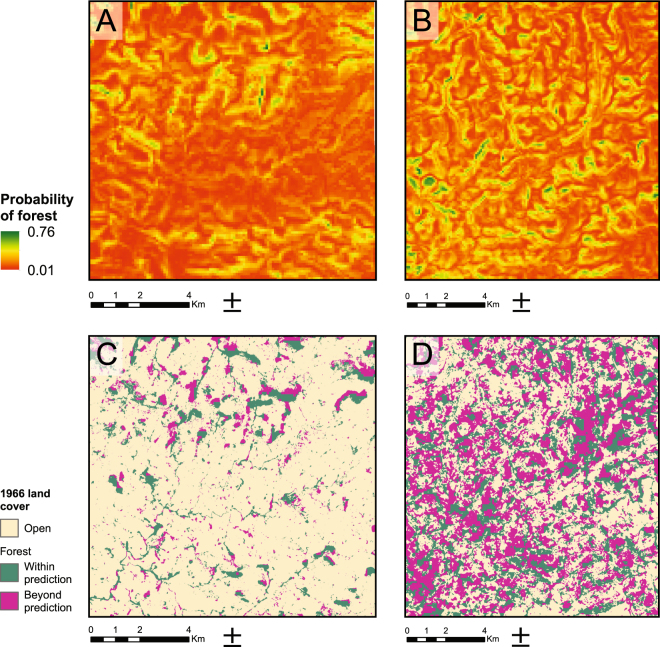
Vegetation models. Above: Predictive model of forest distribution based on bilinear regression using terrain variables (elevation, slope, aspect and topographic position index) in Lages (**A**) and Campo Belo do Sul (**B**). Below: Actual forest extent in 1966 compared to a model based on aspect and topographic position index in Lages (**C**) and Campo Belo do Sul (**D**).

In order to further explore the relationship between terrain and *Araucaria* forest we employ an inductive model, constructed using solely aspect and topographic position index (TPI, see Methods) as predictors. Taking into account the empirical data about forest distribution in undisturbed environments^31^, the natural spatial extent of *Araucaria* forests is modelled by excluding north-facing slopes and hilltops, and the results are compared to 1966 land cover (Fig. 3). In the Lages control region only 6% of the land cover is constituted by forests extending beyond the model. In the archaeological area of Campo Belo do Sul, 33%, equivalent to nearly 7000 hectares of forest, occurs outside of its predicted maximum natural range.

In Campo Belo do Sul, stable forest is established on many northern slopes and plateaus, which we hypothesise to be due to anthropogenic impacts. To test this hypothesis, vegetation histories were assessed against two primary expectations:

1. Under natural conditions vegetation was: i) stable, and ii) spatially defined following the natural distribution model
2. Forested plateaus and north-facing slopes changed from grasslands to forests associated with human activity

Vegetation histories were reconstructed using carbon isotopes of soil from test pits. Soil test pits have a benefit over pollen data when assessing a distinct spatial pattern in that they provide a highly localised signal, whereas pollen provides a homogenised signal representative of vegetation across a larger wind or water borne catchment area. The organic component of soil retains a signature of the photosynthetic pathway employed by the decomposed vegetation from which it was created. This signature can be isotopically tested, with distinct δ^13^C results for C_3_ (tree) versus C_4_ (grass) vegetation cover. Plants following the C_3_ pathway have δ^13^C values ranging from −32 to −22‰. C_4_ plant δ^13^C values range between −21 and −9‰^25^. In the southern Brazilian highlands Dűmig *et al*.^25^ record δ^13^C values of soils of *Araucaria* forest from −27.7 to −22.2‰, and in grasslands soils from −18.7 to −14.3‰. The mean δ^13^C for grasslands with shrubs has an intermediate value of −20.0‰.

For this study, the insoluble humin component of bulk soil samples was analysed for δ^13^C from soil profiles in 5 cm increments from targeted locations. Soils in the research and control area had qualities comparable to those of other soils in the highlands^25,26^, featuring acidic (pH 4.4–6.2) clayey (mean clay content 64.5% w/w) soils. Thirteen soil profiles were sampled, nine from the Campo Belo do Sul region and four from the control transect in the Lages area.

## Results

**Figure 4:**
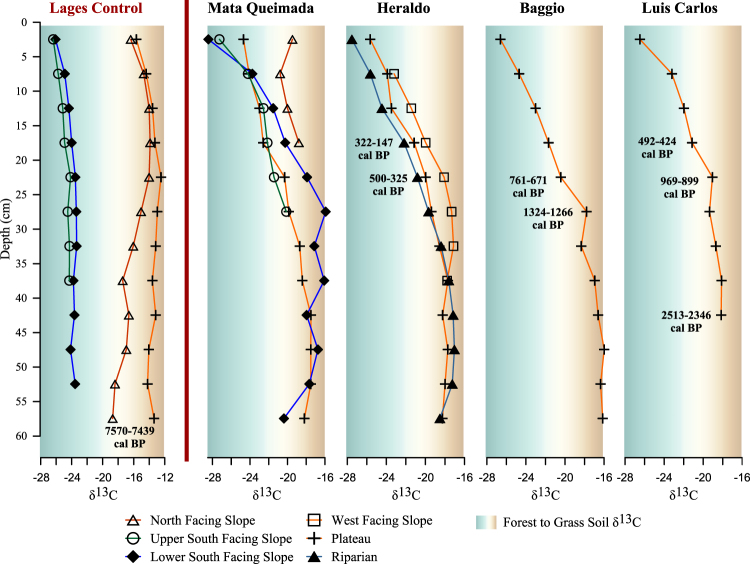
δ^13^C values (‰) of humin from soil test pits for the control region (Lages) and the Campo Belo do Sul transects and targeted plateaus. Profiles are plotted against depth. Background colours are based on δ^13^C values of soil organic matter from soils supporting C_3_ and C_4_ plants as reported by Dümig *et al*.^25^.

As reported for C isotopic soil organic matter profiles elsewhere^25,26^, the shallow layers (0–5 cm) of all profiles are characterised by depleted/enriched δ^13^C values associated to the forest or grassland vegetation cover, respectively (Fig. 4, Supplementary Table S4). In the Lages area, soil profiles were analysed from a grass covered north-facing slope and plateau, and forested lower and upper south-facing slopes, in a control transect corresponding to a natural forest distribution. Isotopic results of soil organic matter confirm that the south-facing slope was predominantly covered by C_3_ plants (probably trees and shrubs) throughout its history with the lower slope δ^13^C ranging between −26.18‰ and -23.45‰, and the upper slope ranging between −26.48‰ and −24.24‰. The plateau showed a 7500 year stable signal ranging between 15.74‰ and −12.56‰ and constituting C_4_ plants. The north-facing slope also showed enriched δ^13^C values (−18.80‰ to −13.96‰), associated with C_4_ grassland vegetation cover, although the δ^13^C value of −18.8‰ at 55–60 cm suggests the dominance of C_4_ plants and the presence of C_3_ plants.

In Campo Belo do Sul, a transect was sampled at Mata Queimada where the north-facing slope is currently covered by grassland with shrubs, and the plateau, as well as the south-facing slope are presently forested. The north-facing slope sample was taken within 50 m of the stream at the base of the slope. The soil was only 20 cm deep to bedrock. The soil displays a stable vegetation history with δ^13^C values ranging from −18.84‰ to −20.84‰, suggesting a mixture of C_3_ and C_4_ plants. The upper south-facing slope also has a shallow profile, with bedrock at 30 cm. The profile shows increasing inputs from C_3_ plants through time. δ^13^C values range from −20.18‰ at 25–30 cm, to −24.27‰ at 5–10 cm, with the more depleted upper 5 cm providing a value of −27.32‰. The lower south-facing slope is particularly interesting. After fluctuating δ^13^C values from −20.43‰ at 55–60 cm, to enriched δ^13^C values of −15.97‰ at 25–30 cm, there is a depletion to −23.81‰ at 5–10 cm, and −28.5 in the top 5 cm. The lower profile suggests a predominantly grassland cover with varying C_3_ inputs from shrubs and trees, before a transition to forest. The increasing C_3_ inputs of the upper slope are matched by the results from the plateau. From 30–35 cm to the surface, a steady increase of C_3_ inputs is recorded (−18.74‰ to −24.75‰), marking a transition from C_4_ grassland to forest on the plateau.

A transect close to the Rio Caveiras at Heraldo sampled the now forested plateau, a forested west-facing slope, and the floodplain. The floodplain is characterised by a narrow band of riparian forest, and a mosaic of small forest patches, grasses, and shrubs. The floodplain profile was sampled within a 50 m wide section of riparian forest and shows a transition to increasing C_3_ inputs beginning around 20–25 cm. From 55–60 cm to 25–30 cm, the area was characterised by a C_4_ signal (mean −17.98‰ ± 0.97). C_3_ inputs in the upper 25 cm changed values to −27.57‰ in the top 5 cm. The profile from the west-facing slope was close to the edge of the limit of predicted natural forest. Bedrock was encountered at 40 cm. The location displays a grassland signal (mean -17.61‰ ± 0.44 between 40 and 20 cm). δ^13^C values transition from 18.12‰ at 20–25 cm (dating to 500–325 cal BP [Supplementary Table S5]) to −20.00‰ at 15–20 cm (322–147 cal BP), with subsequent increase in C_3_ inputs to −23.24‰ at 5–10 cm. A similar transition is recorded on the plateau. From 55–60 cm to 25–30 cm, vegetation history was dominated by C_4_ inputs (average −18.29‰ ± 0.58). δ^13^C values in the upper 25 cm record the replacement of C_4_ *campos* taxa with forest vegetation.

Two further soil profiles tested forested plateaus in close proximity to archaeological sites. Both domestic and public architecture are in the vicinity of the Baggio profile. The profile shows a transition from a C_4_ to a C_3_ dominated landscape. Between 55–60 cm and 25–30 cm the vegetation was largely stable grasslands constituted of C_4_ plants with a mean δ^13^C value of −16.89‰ ± 0.88. In the upper 25 cm δ^13^C values are more depleted, with C_3_ inputs becoming dominant by 15–20 cm (−21.7‰) up to the present (−26.64‰). AMS dating of the insoluble humin provides a date of 1324 to 1266 cal BP at 25–30 cm when the vegetation was stable C_4_ grassland. A date of 761–671 cal BP at 20–25 cm, marks the transition in vegetation.

The profile on the plateau at Luis Carlos is in an area rich with pit houses and mounds. The profile was collected close to a mound structure with both domestic and ritual components. As with the Baggio plateau, the vegetation signature shows a transition from C_4_ plant dominance (45 to 20 cm mean δ^13^C −18.68‰ ± 0.54) to C_3_ plant vegetation cover (−23.21‰ at 5–10 cm), marking a replacement of C_4_ grasslands with forest. At 969–899 cal BP (20–25 cm) the vegetation was probably associated with a mixture of C_4_ and C_3_ plants (−19.06‰), with C_3_ inputs (−21.14‰) having overtaken by 492–424 cal BP (15–20 cm). Activity at the nearby mound coincides with the transition (474-315 cal BP).

## Discussion

Isotopic results (Fig. 4; Supplementary Table S4) corroborate the model of natural vegetation distribution and confirm that human inputs caused the expansion of the forest at the expense of grasslands in Campo Belo do Sul. Before intensive human occupation, the landscape followed the natural distribution model, with forest patches limited to south-facing slopes in a mosaic with C_4_ grasslands. The north-facing and upper south-facing slopes of Mata Queimada both show a history of stable vegetation, characterised by grassland and forest signatures, respectively. However, the plateaus at Mata Queimada, Luis Carlos, Baggio, and Heraldo, as well as the west-facing slope of Heraldo, expected grasslands under the model of natural vegetation distribution, show a transition in dominant vegetation inputs from the C_4_ (grassland) signal to C_3_ (forest).

This replacement of grasslands with forest coincides with past human activity indicated by the SCPD (Fig. 2). Vegetation was stable up to the time of initial permanent settlement of the region, with C_4_ grasses present on plateaus at Baggio (ca. 1324–1266 cal BP) and Luis Carlos (ca. 969–899 cal BP). Local settlement began ca. 1000 cal BP, followed by a wave of expansion ca. 850 cal BP, reaching its peak ca. 500 cal BP. Vegetation transitions coincided with human activity at Luis Carlos, and at Baggio, by ca. 761–671 cal BP, the C_3_ signal had already begun to replace C_4_ inputs, confirming that the forest expansion was occurring during active occupation of the region, and not as a forest invasion of the depopulated post-collapse landscape.

**Figure 5:**
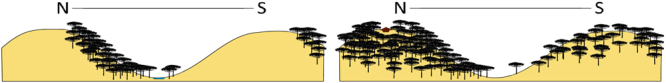
Representative model of forest distribution under undisturbed conditions (left) in which forest is naturally restricted to south-facing slopes, and in areas of archaeological activity (right) in which forest has expanded across the landscape.

Minor expansions of forest around 4000 cal BP, recorded in the regional pollen records, were likely facilitated by the onset of the more stable wetter climate recorded in the speleothem proxy. Per Behling *et al*.^2^, forests likely spread along streams into a network of gallery forest; however, this expansion was spatially limited by topographic features as predicted by the vegetation model (Fig. 5). The onset of the rapid regional expansion of the forest, between ca. 1410 and 900 cal BP, actually occurs during a relatively dry/less humid period that coincides with increases in archaeological activity as demonstrated by the SCPDs (Fig. 2). Regional archaeological activity had reached its peak by the onset of the wetter Little Ice Age, before European contact instigated dramatic indigenous cultural decline across the highlands. Various authors^3,5,6^ have argued for an anthropogenic origin for forest expansion over a climatic response due to the close spatial relationship between cultural activity and the distribution of *Araucaria* forest, as well as the demand for an agent for longer-distance dispersal of the heavy seed. These hypotheses had not been adequately tested integrating archaeology, paleoecology, and paleoclimate data. We also add spatial modelling to confirm and refine this hypothesis by defining the natural geographic boundaries of forest distribution and the necessity for human agents for seed success beyond these boundaries.

Despite cultural collapses in the region after European contact, continued depleted δ^13^C values of the soil organic matter up to the modern day demonstrate the lasting impacts of anthropogenic inputs on vegetation succession. Once present, the new vegetation regime alters understory dynamics, seed recruitment, edaphic qualities, moisture availability and light regime to become self-supporting and subject to a new successional trajectory^31^. Subsequent climatic fluctuations, including prolonged relatively dry periods, did not result in forest dieback or grassland expansion, even after regional population declines had drastically reduced any direct anthropogenic management until 20th century logging.

Importantly, regardless of climatic fluctuations, vegetation patterns in the control transect correspond with the natural distribution model and remain stable up to the present day. Climatic fluctuations, including a sustained wet period (the wettest in the history of the area) ca. 2000–1750 cal BP, were not enough to cause a vegetation response that could overcome geographic boundaries to vegetation distribution.

Distinguishing long-term human and climate impacts on vegetation dynamics is of critical importance considering the global significance of this biodiversity hotspot and the endangered status of *A. angustifolia* and the *Araucaria* forest. An integrated paleoecological and archaeological approach sheds light on the processes and dynamics that created the modern vegetation distribution, revealing the structure of current ecosystems to be a function of centuries of past human land management. The data presented here provide evidence of a millennia of sustainable resource use that not only incorporated the *Araucaria* forest into the core of the indigenous economy, but actually expanded forest beyond natural boundaries of habitat distribution. Conservation strategies that exclude human land-use may therefore be misguided and counterproductive when balancing cultural heritage, economic development, and conservation goals^35^.

## Methods

### Predictive Vegetation Distribution Model

We used binomial logistic regression to generate a predictive model of *Araucaria* forest distribution under natural conditions, with forest presence as the outcome variable and terrain parameters as predictors. The model was based on a 10 × 10 km control area south of Lages, where relatively undisturbed conditions were observed. Forest distribution was assessed from 1966 satellite imagery (CORONA) with a resolution of 4 m (available at https://earthexplorer.usgs.gov/). We manipulated the images in ArcGIS 10.2, where the Maximum Likelihood Classification tool was used to convert them into rasters of forest presence (1) or absence (0). The raster was converted into a grid of points to extract the values of the terrain predictor variables. Slope and aspect were generated from elevation data (SRTM with a resolution of 90 m available at https://earthexplorer.usgs.gov/) using the Spatial Analyst toolset in ArcGIS 10.2. Aspect was reclassified into south-facing (1) and facing any other direction (0). Topographic position index (TPI) is a measure of deviation of a terrain cell from the average elevation of its surroundings within a predetermined radius, with positive TPI indicating ridges and negative TPI indicating valleys. TPI was calculated from the SRTM raster using the Custom Land Facet Analysis toolset (available at http://www.jennessent.com/arcgis/land_facets.htm) with the recommended search radius of 5 cells. All terrain rasters were normalised so that the model could be generalised to areas with different elevation ranges from that of the control area. The logistic regression was run in R 3.3.3 and the coefficients were used in the Raster Calculator tool of ArcGIS 10.2 to generate a surface of forest probability. For the second model of *Araucaria* natural distribution, the TPI raster was reclassified using the recommended values of −6 for valleys and +6 for ridges (http://www.jennessent.com/arcgis/land_facets.htm). The result was added to the aspect raster. North-facing slopes and plateaus were predicted as grasslands (0) whereas south-facing slopes and valleys were coded as potential areas of forest (1) under natural conditions.

### Test pits

Thirteen soil test pits were sampled in 5 cm increments, to a depth of 60 cm or bedrock. Samples from Mata Queimada and the Lages control were collected from transects across valleys, with soil profiles collected from the north-facing slope, the lower south-facing slope, the upper south-facing slope, and the plateau. Heraldo was also collected as transect, minus the forested north-facing slope due to the width of the Rio Caveiras. Single profiles were collected from Baggio and Luis Carlos on forested plateaus that are directly related to archaeological sites.

### Isotopes and AMS Dating

Following protocol described by Pessenda *et al*.^36,37^, the soil organic matter from physical pre-treated bulk samples of each 5 cm increment were analysed for δ^13^C and Total Organic Carbon (TOC) at the Stable Isotope Laboratory of Centre for Nuclear Energy in Agriculture (CENA), University of São Paulo (Supplementary Table S4). Organic carbon is expressed as percentage of dry weight and ^13^C results are given as δ^13^C with respect to VPDB standard using the conventional δ (‰) notations:

$${\delta }^{13}{\rm{C}}(\textperthousand )=[({{\rm{R}}}_{{\rm{s}}{\rm{a}}{\rm{m}}{\rm{p}}{\rm{l}}{\rm{e}}}/{{\rm{R}}}_{{\rm{s}}{\rm{t}}{\rm{a}}{\rm{n}}{\rm{d}}{\rm{a}}{\rm{r}}{\rm{d}}}){\textstyle \,\text{-}\,}1]1000$$

where R_sample_ and R_standard_ are the ^13^C/^12^C ratios of the sample and standard, respectively. Analytical precision is ±0.2‰.

The ^14^C analyses on soil humin^37^ fraction were carried out by Accelerator Mass Spectrometry (AMS) by Beta Analytic. Radiocarbon ages are expressed as cal (2σ) BP (Before AD 1950) normalised to δ^13^C of −25‰ VPDB and as calibrate cal (2σ) BP^38^.

Radiocarbon dating of Soil Organic Matter (SOM) is complicated. Studies have shown that different components of SOM have different ages^36,37,39^. Comparison of ^14^C dating of humin and total soil fraction (three SOM fractions) from distinct soils, under different vegetation cover, in several regions of Brazil^36,37,39^, showed the insoluble humin fraction to be older, indicating contamination of total SOM by younger carbon in the fulvic and humic acids. The ^14^C ages of associated charcoal, in most cases, are in agreement with the humin fraction, or 20% older in average^39^. The test studies show that in the absence of charcoal, the humin fraction is a reliable material for ^14^C dating in soils. However, the humin fraction ages could be assumed as the minimum ages for carbon in soils.

The chronology for the LC core relies on three ^14^C dates in an age-depth model constructed in Bacon v2.2 within R. Bulk-sediment soil profile material was collected for conventional AMS radiocarbon dating. Radiocarbon ages were calibrated within Bacon using SHCal13 and modelled using Student-t test distributions with wide tails to negate the need of identifying and removing potential outliers in the age-depth model. Age-depth model mean accumulation rate priors in Bacon were calculated using the ^14^C chronology (acc.mean = 58 and memory priors were set slightly below default so that the model would capture accumulation rate changes driven by variable sediment delivery from the catchment (mem.strength = 2; mem.mean = 0.3). Model means and 2σ age distributions were calculated from Markov chain Monte Carlo age-depth iterations through the soil profile.

### Radiocarbon Dates & SCPD

The Sum of the Calibrated Probability Distributions (SCPD or SPD) is a standard method for representing chronological trends in radiocarbon datasets. SCPDs are produced by calibrating each independent date in the sample and adding the results to produce a single density distribution. This has the advantage of including the full range of probabilities associated with calibrated dates, instead of using single point estimates^40–44^. SCPDs were built in OxCal using the Sum function and the ShCal13 calibration curve^45,46^. Dates clearly associated with southern Jê contexts were compiled from previously published syntheses, recently published papers, books, unpublished academic theses, and site reports (Supplementary Table S2), to which we added 42 new dates from our own field work (Supplementary Table S1). The complete dataset, before filtering, contains 245 dates from 118 archaeological sites. We applied measures of chronometric hygiene to gain more precision, overcome fluctuations in the calibration curve, and obtain a dataset that was as reliable as possible; excluding dates that: i) had a standard error equal to or larger than 100 years; ii) whose provenance was unclear; or iii) not assigned to a specific laboratory number. Additionally, to account for oversampling of some sites and phases within those sites, we applied a binning procedure^41–43^. Dates within sites were ordered and grouped into 100-year bins. Dates within each bin were merged with the R_combine function of OxCal. Timpson *et al*.^43^ found that different values for the bin-width did not affect the final shape of the SCPD. This procedure is necessary because a sum of the calibrated dates assumes that observations are independent, whereas this is not the case when multiple dates were obtained for single sites or phases within them. The final filtered dataset contained 134 dates. Despite the decrease in sample size, the filtered SCPD is highly correlated with an SCPD built with all available radiocarbon dates (Pearson’s r2 = 0.97). In addition to the SCPD, we plotted the frequencies of sites by 100 year bins using the median of the calibrated dates, each site being counted only once per time bin.

### Data and materials availability

All data needed to evaluate the conclusions in the paper are present in the paper and/or the Supplementary Materials. Additional data related to this paper may be requested from the authors.

## Electronic supplementary material

Supplementary Information
