# Supplementary material for: Uncoupling human and climate drivers of late Holocene vegetation change in southern Brazil
Source: Sci Rep. 2018 May 17;8:7800. doi: 10.1038/s41598-018-24429-5 (PMC5958110; doi:10.1038/s41598-018-24429-5)
Supplement: Supplementary file 1 — Supplementary Information [file 41598_2018_24429_MOESM1_ESM.docx]

**Uncoupling human and climate drivers of late Holocene vegetation change in southern Brazil**

Mark Robinson, Jonas Gregorio De Souza, S. Yoshi Maezumi, Macarena Cárdenas, Luiz Pessenda, Keith Prufer, Rafael Corteletti, Deisi Scunderlick, Francis Edward Mayle, Paulo De Blasis, José Iriarte

**Supplementary Information**

Table S1. AMS radiocarbon dates from the Campo Belo do Sul region

| Site | Type | Structure | Context | RCYBP | δ13C‰ | Laboratory number |
| --- | --- | --- | --- | --- | --- | --- |
| Abreu & Garcia | MEC | Mound B | Cremated deposit | 330 ± 20 | -24.5 | UGAMS-19003 |
| Abreu & Garcia | MEC | Mound B | Cremated deposit | 360 ± 30 | -24.9 | Beta-395741 |
| Abreu & Garcia | MEC | Mound A | Cremated deposit #12 | 230 ± 30 | -27.8 | Beta-395740 |
| Abreu & Garcia | MEC | Mound A | Cremated deposit #11 | 270 ± 30 | -25.7 | Beta-395743 |
| Abreu & Garcia | MEC | Mound A | Cremated deposit #16 | 370 ± 30 | -26.4 | Beta-395744 |
| Abreu & Garcia | MEC | Mound A | Cremated deposit #6 | 390 ± 30 | -24.1 | Beta-417389 |
| Abreu & Garcia | MEC | Mound A | Cremated deposit #14 | 400 ± 30 | -23.4 | Beta-395742 |
| Abreu & Garcia | MEC | Mound A | Burnt feature | 300 ± 30 | -23.3 | Beta-414096 |
| Baggio 1 | Pit house | External area | Firepit | 390 ± 30 | -24.1 | Beta-414094 |
| Baggio 1 | Pit house | Mound A | Mound fill | 600 ± 30 | -22 | Beta-438293 |
| Baggio 1 | Pit house | External area | Firepit | 840 ± 30 | -26.9 | Beta-414095 |
| Baggio 1 | Pit house | House 11 | Floor #5 | 170 ± 30 | -25.8 | Beta-438290 |
| Baggio 1 | Pit house | House 11 | Floor #3 | 300 ± 30 | -24.2 | Beta-438291 |
| Baggio 1 | Pit house | House 11 | Floor #1 | 330 ± 30 | -27.2 | Beta-438290 |
| Baggio 1 | Pit house | House 3 | Floor #9 | 440 ± 30 | -26.9 | Beta-438286 |
| Baggio 1 | Pit house | House 3 | Floor #8 | 550 ± 30 | -21.8 | Beta-438287 |
| Baggio 1 | Pit house | House 3 | Floor #3 | 330 ± 30 | -27.4 | Beta-438289 |
| Baggio 1 | Pit house | House 3 | Firepit on floor #1 | 320 ± 30 | -27.7 | Beta-438288 |
| Baggio 1 | Pit house | House 2 | Floor #2 | 360 ± 30 | -22.5 | Beta-414092 |
| Baggio 1 | Pit house | House 2 | Burning on floor #1 | 320 ± 30 | -26.6 | Beta-414093 |
| Baggio 1 | Pit house | House 1 | Firepit on floor #12 | 280 ± 30 | -25.2 | Beta-414080 |
| Baggio 1 | Pit house | House 1 | Floor #11 | 340 ± 30 | -23.5 | Beta-414081 |
| Baggio 1 | Pit house | House 1 | Floor #10 | 350 ± 30 | -28.9 | Beta-414082 |
| Baggio 1 | Pit house | House 1 | Floor #9 | 360 ± 30 | -27 | Beta-414091 |
| Baggio 1 | Pit house | House 1 | Floor #8 | 520 ± 30 | -22.6 | Beta-414083 |
| Baggio 1 | Pit house | House 1 | Floor #7 | 350 ± 30 | -24.7 | Beta-414084 |
| Baggio 1 | Pit house | House 1 | Burnt roof on floor #5 | 340 ± 30 | -27.4 | Beta-414085 |
| Baggio 1 | Pit house | House 1 | Burnt roof on floor #4 | 860 ± 30 | -23.8 | Beta-414086 |
| Baggio 1 | Pit house | House 1 | Burnt roof on floor #3 | 300 ± 30 | -26.3 | Beta-414087 |
| Baggio 1 | Pit house | House 1 | Burnt roof on floor #2 | 460 ± 30 | -24.1 | Beta-414088 |
| Baggio 1 | Pit house | House 1 | Burnt roof on floor #1 | 630 ± 30 | -24.8 | Beta-414089 |
| Baggio 2 | Pit house | House 1 | Floor #2 | 630 ± 30 | -26.2 | Beta-436322 |
| Baggio 2 | Pit house | House 1 | Firepit on floor #1 | 660 ± 30 | -25 | Beta-436323 |
| Darci | Pit house |  |  | 550 ± 30 | -26.8 | Beta-460413 |
| DiCarli | Pit house | House 1 | Firepit on floor #2 | 460 ± 30 | -25.5 | Beta-436324 |
| Edineia | Pit house |  |  | 390 ± 30 | -27.1 | Beta-460416 |
| João 3 | Pit house | House 2 | Floor #7 | 270 ± 30 | -28.6 | Beta-436319 |
| João 3 | Pit house | House 2 | Floor #6 | 960 ± 30 | -27.8 | Beta-436318 |
| João 3 | Pit house | House 2 | Floor #4 | 400 ± 30 | -26.6 | Beta-436316 |
| João 3 | Pit house | House 2 | Floor #3 | 380 ± 30 | -27.5 | Beta-436317 |
| João 3 | Pit house | House 2 | Floor #1 | 1020 ± 30 | -26.6 | Beta-436316 |
| Luís Carlos 1 | Mound |  | Burnt surface | 370 ± 30 | -25.1 | Beta-471036 |
| Luís Carlos 1 | Mound |  |  | 370 ± 30 | -22.8 | Beta-460414 |
| Manno | Pit house | House 1 | Firepit on floor #2 | 450 ± 30 | -29.8 | Beta-436320 |
| Pedro 1 | MEC | Mound 1 | Mound fill | 640 ± 30 | -25.1 | Beta-436321 |
| Travessão | Pit house | House 5 | Firepit on floor #1 | 560 ± 30 | -26.1 | Beta-417390 |
| Valdomiro | MEC |  |  | 120 ± 30 | -26.1 | Beta-460412 |

Table S2. All radiocarbon dates from southern proto Jê contexts.

| Site | RCYBP | Laboratory number | Type | Reference |
| --- | --- | --- | --- | --- |
| Abrigo da Janela | 1790 ± 210 | ANU-19227 | Rock shelter | Parellada (2005) |
| Alfredo Wagner | 1720 ± 40 | CAMS-53916 | Rock shelter | De Masi (2001) |
| Apucarana | 590 ± 40 | Beta-238714 | Surface site | Schmitz and Rogge (2008) |
| Areia Branca 5 | 880 ± 60 | Beta-24751 | Surface site | Araujo (2001) |
| Areia Branca 6 | 1530 ± 40 | Beta-35824 | Mound | Araujo (2001) |
| Areia Branca 6 | 1430 ± 60 | Beta-24752 | Mound | Araujo (2001) |
| Arroio da Cruz I | 1160 ± 40 | Beta-211732 | Surface site | De Blasis et al. (2014) |
| Arroio da Cruz I | 1080 ± 60 | Beta-209703 | Surface site | De Blasis et al. (2014) |
| Base Aérea | 800 ± 70 | SI-243 | Shell mound | Rohr (1960) |
| Bonin 1 | 1210 ± 25 | UGAMS- 25384 | Pit house | Unpublished |
| Bonin 1 | 640 ± 40 | Beta-298215 | Pit house | Corteletti (2012) |
| Bonin 1 | 610 ± 50 | Beta-298216 | Pit house | Corteletti (2012) |
| Bonin 1 | 360 ± 20 | UGAMS- 25385 | Pit house | Unpublished |
| Bonin 1 | 340 ± 25 | UGAMS- 25388 | Pit house | Unpublished |
| Bonin 1 | 330 ± 25 | UGAMS- 25386 | Pit house | Unpublished |
| Bonin 1 | 310 ± 25 | UGAMS- 25383 | Pit house | Unpublished |
| Bonin 1 | 310 ± 25 | UGAMS- 25387 | Pit house | Unpublished |
| BS19 | 595 ± 50 | Gif-10040 | Surface site | De Blasis (1996) |
| Caieira | 710 ± 95 | Isotopes 2624 | Shell mound | De Blasis et al. (2014) |
| Campos de Lages | 1117 ± 80 | N/A | Pit house | Schmitz and Brochado (1972) |
| Cel. Passos Maia | 1140 ± 30 | N/A | Pit house | Schwengber et al. (2012) |
| Fazenda Marrecas IV | 890 ± 180 | N/A | Surface site | Parellada (2005) |
| Galheta IV | 1360 ± 40 | Beta-280010 | Mound | De Blasis et al. (2014) |
| Galheta IV | 1070 ± 40 | Beta-280011 | Mound | De Blasis et al. (2014) |
| Galheta IV | 980 ± 40 | Beta-211734 | Mound | De Blasis et al. (2014) |
| Galheta IV | 950 ± 40 | Beta-28012 | Mound | De Blasis et al. (2014) |
| Itá | 750 ± 30 | N/A | Surface site | Farias and Schmitz (2013) |
| Marechal Luz | 880 ± 100 | M-1202 | Shell mound | Bryan (1965) |
| P. Costão do Ilhote | 980 ± 40 | Beta-211733 | Shell mound | De Blasis et al. (2014) |
| PM-01 | 760 ± 60 | Beta-221418 | Mound and enclosure | Iriarte et al. (2008) |
| PM-01 | 760 ± 40 | Beta-237106 | Mound and enclosure | Iriarte et al. (2008) |
| PM-01 | 720 ± 40 | Beta-237105 | Mound and enclosure | Iriarte et al. (2008) |
| PM-01 | 480 ± 60 | Beta-221417 | Mound and enclosure | Iriarte et al. (2008) |
| Posto Fiscal | 1070 ± 40 | Beta-303594 | Mound and enclosure | Iriarte et al. (2008) |
| Posto Fiscal | 370 ± 40 | Beta-309037 | Mound and enclosure | Iriarte et al. (2008) |
| Posto Fiscal | 330 ± 40 | Beta-304479 | Mound and enclosure | Iriarte et al. (2008) |
| Posto Fiscal | 200 ± 30 | Beta-309038 | Mound and enclosure | Iriarte et al. (2008) |
| PR-CT-53 | 848 ± 70 | Beta-22644 | Pit house | Chmyz (1995) |
| PR-CT-53 | 558 ± 50 | Beta-22646 | Pit house | Chmyz (1995) |
| PR-CT-93 | 940 ± 70 | Beta-180907 | Pit house | Chmyz et al. (2003) |
| PR-CT-93 | 850 ± 50 | Beta-180907 | Pit house | Chmyz et al. (2003) |
| PR-CT-93 | 680 ± 70 | Beta-180905 | Pit house | Chmyz et al. (2009) |
| PR-CT-93 | 660 ± 60 | Beta-180904 | Pit house | Chmyz et al. (2003) |
| PR-CT-93 | 580 ± 60 | Beta-180903 | Pit house | Chmyz et al. (2003) |
| PR-MN-04 | 595 ± 60 | SI-6396 | Surface site | Chmyz et al. (2008) |
| PR-SM-14 | 490 ± 40 | Beta-256208 | Surface site | Chmyz et al. (2009) |
| PR-SM-16 | 1150 ± 40 | Beta-256211 | Surface site | Chmyz et al. (2009) |
| PR-SM-17 | 1030 ± 50 | Beta-256210 | Surface site | Chmyz et al. (2009) |
| PR-SM-17 | 920 ± 40 | Beta-256209 | Surface site | Chmyz et al. (2009) |
| PR-UB-4 | 855 ± 95 | SI-2193 | Pit house | Chmyz (1981) |
| PR-UB-4 | 735 ± 95 | SI-2194 | Pit house | Chmyz (1981) |
| PR-UB-4 | 470 ± 95 | SI-2192 | Pit house | Chmyz (1981) |
| PR-UV-1 | 800 ± 50 | SI-141 | Rock shelter | Chmyz (1968) |
| PR-UV-11 | 680 ± 70 | SI-1010 | Mound and enclosure | Schmitz (1988) |
| PR-UV-12 | 810 ± 90 | SI-892 | Pit house | Schmitz (1988) |
| PR-UV-12 | 623 ± 120 | SI-691 | Pit house | Schmitz (1988) |
| PR-UV-12 | 255 ± 100 | SI-692 | Pit house | Schmitz (1988) |
| PR-UV-17 | 1475 ± 65 | SI-2197 | Rock shelter | Chmyz (1981) |
| PR-UV-38 | 190 ± 75 | SI-5013 | Surface site | Chmyz (1981) |
| PR-UV-47 | 1635 ± 100 | SI-5014 | Surface site | Chmyz et al. (2009) |
| PR-UV-48 | 1420 ± 50 | Beta-22647 | Surface site | Chmyz et al. (2009) |
| Rio do Meio | 780 ± 60 | Beta-178077 | Surface site | Fossari (2004) |
| RS-127 | 1480 ± 70 | SI-603 | Pit house | Schmitz (1969) |
| RS-127 | 1330 ± 100 | SI-605 | Pit house | Schmitz (1969) |
| RS-127 | 1140 ± 40 | SI-602 | Pit house | Schmitz (1969) |
| RS-127 | 840 ± 60 | SI-606 | Pit house | Schmitz (1969) |
| RS-127 | 630 ± 70 | SI-604 | Pit house | Schmitz (1969) |
| RS-40 | 1520 ± 90 | SI-607 | Pit house | Schmitz (1969) |
| RS-68 | 620 ± 90 | SI-608 | Pit house | Schmitz (1969) |
| RS-A-2 | 1515 ± 105 | SI-805 | Pit house | Schmitz (1988) |
| RS-A-2 | 1385 ± 95 | SI-806 | Pit house | Schmitz (1988) |
| RS-A-2 | 970 ± 95 | SI-808 | Pit house | Schmitz (1988) |
| RS-A-27 | 870 ± 60 | Beta-144246 | Pit house | Schmitz et al. (2002) |
| RS-A-27 | 870 ± 50 | Beta-144244 | Pit house | Schmitz et al. (2002) |
| RS-A-27 | 520 ± 60 | Beta-144245 | Pit house | Schmitz et al. (2002) |
| RS-A-27 | 40 ± 60 | Beta-144247 | Pit house | Schmitz et al. (2002) |
| RS-A-27 | 30 ± 50 | Beta-144243 | Pit house | Schmitz et al. (2002) |
| RS-A-29 | 710 ± 60 | Beta-178090 | Pit house | Schmitz et al. (2002) |
| RS-A-29 | 680 ± 80 | Beta-153842 | Pit house | Schmitz et al. (2002) |
| RS-A-29 | 380 ± 60 | Beta-153843 | Pit house | Schmitz et al. (2002) |
| RS-A-29 | 370 ± 50 | Beta-178089 | Pit house | Schmitz et al. (2002) |
| RS-A-8 | 700 ± 60 | N/A | Pit house | Schmitz (1988) |
| RS-AN-03 | 1070 ± 70 | Beta-178135 | Pit house | Copé (2006) |
| RS-AN-03 | 1000 ± 40 | Beta-166588 | Pit house | Copé (2006) |
| RS-AN-03 | 880 ± 40 | Beta-183020 | Pit house | Copé (2006) |
| RS-AN-03 | 870 ± 50 | Beta-183022 | Pit house | Copé (2006) |
| RS-AN-03 | 780 ± 60 | Beta-178136 | Pit house | Copé (2006) |
| RS-AN-03 | 690 ± 60 | Beta-183021 | Pit house | Copé (2006) |
| RS-AN-03 | 550 ± 40 | Beta-166584 | Pit house | Copé (2006) |
| RS-AN-03 | 370 ± 50 | Beta-166584 | Pit house | Copé (2006) |
| RS-AN-03 | 250 ± 50 | Beta-178134 | Pit house | Copé (2006) |
| RS-AN-03 | 80 ± 50 | Beta-166586 | Pit house | Copé (2006) |
| RS-C-12 | 630 ± 205 | SI-1201 | Rock shelter | Ribeiro (1974) |
| RS-C-14 | 745 ± 65 | SI-1198 | Rock shelter | Ribeiro (1974) |
| RS-P-12 | 1810 ± 85 | SI-813 | Surface site | Schmitz and Brochado (1972) |
| RS-P-27 | 950 ± 80 | SI-812 | Surface site | Schmitz and Brochado (1972) |
| RS-PE-10a | 465 ± 40 | SI-6558 | Pit house | Ribeiro and Ribeiro (1985) |
| RS-PE-10a | 390 ± 50 | SI-6556 | Pit house | Ribeiro and Ribeiro (1985) |
| RS-PE-10b | 355 ± 50 | SI-6559 | Pit house | Ribeiro and Ribeiro (1985) |
| RS-PE-11 | 1200 ± 40 | N/A | Pit house | Iriarte et al. (2013) |
| RS-PE-21 | 350 ± 40 | Beta-242868 | Mound and enclosure | De Souza and Copé (2010) |
| RS-PE-26a | 635 ± 45 | SI-6561 | Pit house | Ribeiro and Ribeiro (1985) |
| RS-PE-28a | 650 ± 55 | SI-6563 | Pit house | Ribeiro and Ribeiro (1985) |
| RS-PE-28a | 420 ± 55 | SI-6562 | Pit house | Ribeiro and Ribeiro (1985) |
| RS-PE-29 | 490 ± 40 | Beta-242869 | Mound and enclosure | De Souza and Copé (2010) |
| RS-PE-29 | 340 ± 40 | Beta-242860 | Mound and enclosure | De Souza and Copé (2010) |
| RS-PE-31 | 110 ± 40 | Beta-276193 | Mound and enclosure | Iriarte et al. (2013) |
| RS-PE-41 | 1140 ± 40 | N/A | Pit house | Iriarte et al. (2013) |
| RS-PF-01 | 1300 ± 70 | SI-601 | Surface site | Schmitz (1988) |
| RS-RP-164b | 915 ± 145 | SI-4066 | Pit house | Ribeiro (1980) |
| RS-S-282 | 1380 ± 110 | SI-414 | Surface site | Brochado et al. (1969) |
| RS-S-328 | 1655 ± 65 | N/A | Rock shelter | Brochado et al. (1969) |
| RS-S-359 | 1740 ± 65 | N/A | Rock shelter | Brochado et al. (1969) |
| RS-S-61 | 1190 ± 100 | SI-409 | Surface site | Brochado et al. (1969) |
| RS-T-123 | 1040 ± 30 | Beta-343953 | Pit house | Wolf et al. (2016) |
| RS-T-123 | 970 ± 30 | Beta-385781 | Pit house | Wolf et al. (2016) |
| RS-T-123 | 940 ± 30 | Beta-343954 | Pit house | Wolf et al. (2016) |
| RS-T-126 | 1140 ± 30 | Beta-385782 | Pit house | Wolf et al. (2016) |
| RS-T-130 | 840 ± 30 | Beta-423195 | Surface site | Wolf et al. (2016) |
| RS-VZ-25 | 400 ± 100 | SI-600 | Surface site | Miller (1969) |
| RS-VZ-43 | 830 ± 60 | SI-598 | Surface site | Miller (1969) |
| RS-VZ-44 | 160 ± 70 | SI-599 | Surface site | Miller (1969) |
| Sao Joaquim | 1085 ± 80 | N/A | Pit house | Farias and Schmitz (2013) |
| SC-AB-04 | 400 ± 40 | Beta-190292 | Pit house | De Masi (2005) |
| SC-AB-04 | 370 ± 40 | Beta-190293 | Pit house | De Masi (2005) |
| SC-AB-48 | 450 ± 40 | Beta-190294 | Surface site | De Masi (2005) |
| SC-AB-92 | 190 ± 40 | Beta-190296 | Pit house | De Masi (2005) |
| SC-AB-93 | 690 ± 40 | Beta-190301 | Pit house | De Masi (2005) |
| SC-AB-93 | 650 ± 40 | Beta-190297 | Pit house | De Masi (2005) |
| SC-AB-93c | 300 ± 40 | Beta-190298 | Pit house | De Masi (2005) |
| SC-AB-93d | 840 ± 40 | Beta-190300 | Pit house | De Masi (2005) |
| SC-AB-93d | 340 ± 40 | Beta-190299 | Pit house | De Masi (2005) |
| SC-AB-95b | 1230 ± 40 | Beta-190302 | Pit house | De Masi (2005) |
| SC-AB-96 | 360 ± 40 | Beta-190303 | Mound and enclosure | De Masi (2005) |
| SC-AG-100 | 390 ± 50 | Beta-226124 | Mound and enclosure | Müller (2008) |
| SC-AG-107 | 970 ± 60 | N/A | Pit house | Müller (2007) |
| SC-AG-107 | 880 ± 70 | N/A | Pit house | Müller (2007) |
| SC-AG-107 | 750 ± 40 | N/A | Pit house | Müller (2007) |
| SC-AG-107 | 720 ± 60 | N/A | Pit house | Müller (2007) |
| SC-AG-107 | 510 ± 40 | N/A | Pit house | Müller (2007) |
| SC-AG-107 | 420 ± 60 | N/A | Pit house | Müller (2007) |
| SC-AG-108 | 350 ± 40 | Beta-226125 | Mound and enclosure | Müller (2008) |
| SC-AG-12 | 690 ± 40 | Beta-185443 | Mound and enclosure | De Masi (2005) |
| SC-AG-12 | 600 ± 40 | Beta-190304 | Mound and enclosure | De Masi (2005) |
| SC-AG-12 | 470 ± 40 | Beta-185444 | Mound and enclosure | De Masi (2005) |
| SC-AG-12 | 430 ± 40 | Beta-185442 | Mound and enclosure | De Masi (2005) |
| SC-AG-18 | 180 ± 40 | Beta-190306 | Surface site | De Masi (2005) |
| SC-AG-40 | 180 ± 40 | N/A | Surface site | Saldanha (2005) |
| SC-AG-75 | 980 ± 40 | Beta-190309 | Mound and enclosure | De Masi (2005) |
| SC-AG-76 | 1050 ± 40 | Beta-190308 | Pit house | De Masi (2005) |
| SC-AG-76 | 940 ± 40 | Beta-190310 | Pit house | De Masi (2005) |
| SC-AG-77 | 420 ± 40 | Beta-190311 | Mound and enclosure | De Masi (2005) |
| SC-AG-98 | 350 ± 40 | Beta-175188 | Mound and enclosure | Müller (2008) |
| SC-CL-10 | 330 ± 90 | N/A | Pit house | Schmitz (1988) |
| SC-CL-43 | 640 ± 40 | Beta-275575 | Pit house | Schmitz et al. (2013a) |
| SC-CL-43 | 470 ± 50 | Beta-256216 | Pit house | Schmitz et al. (2013a) |
| SC-CL-43 | 370 ± 40 | Beta-285996 | Pit house | Schmitz et al. (2013a) |
| SC-CL-43a | 590 ± 40 | Beta-242152 | Pit house | Schmitz et al. (2013a) |
| SC-CL-46 | 910 ± 30 | Beta-357352 | Pit house | Schmitz et al. (2013a) |
| SC-CL-46 | 610 ± 30 | Beta--357351 | Pit house | Schmitz et al. (2013a) |
| SC-CL-46 | 580 ± 30 | Beta-351739 | Pit house | Schmitz et al. (2013a) |
| SC-CL-46 | 510 ± 30 | Beta-357346 | Pit house | Schmitz et al. (2013a) |
| SC-CL-50 | 910 ± 30 | Beta-351740 | Pit house | Schmitz et al. (2013a) |
| SC-CL-51 | 320 ± 30 | Beta-351741 | Pit house | Schmitz et al. (2013a) |
| SC-CL-52 | 870 ± 30 | Beta-351742 | Pit house | Schmitz et al. (2013a) |
| SC-CL-52 | 860 ± 30 | Beta-357350 | Pit house | Schmitz et al. (2013a) |
| SC-CL-56 | 830 ± 40 | Beta-242151 | Pit house | Schmitz et al. (2013a) |
| SC-CL-70 | 1400 ± 40 | Beta-297431 | Pit house | Schmitz et al. (2013b) |
| SC-CL-70 | 1320 ± 40 | Beta-293588 | Pit house | Schmitz et al. (2013b) |
| SC-CL-70 | 1320 ± 40 | Beta-293589 | Pit house | Schmitz et al. (2013b) |
| SC-CL-70 | 1250 ± 40 | Beta-297430 | Pit house | Schmitz et al. (2013b) |
| SC-CL-70 | 1190 ± 40 | Beta-293590 | Pit house | Schmitz et al. (2013b) |
| SC-CL-70 | 1110 ± 40 | Beta-293591 | Pit house | Schmitz et al. (2013b) |
| SC-CL-70 | 1080 ± 30 | Beta-297429 | Pit house | Schmitz et al. (2013b) |
| SC-CL-70 | 470 ± 50 | Beta-297432 | Pit house | Schmitz et al. (2013b) |
| SC-CL-71 | 1360 ± 30 | Beta-319363 | Pit house | Schmitz et al. (2013b) |
| SC-CL-71 | 1330 ± 30 | Beta-319370 | Pit house | Schmitz et al. (2013b) |
| SC-CL-71 | 1310 ± 30 | Beta-319374 | Pit house | Schmitz et al. (2013b) |
| SC-CL-71 | 1290 ± 30 | Beta-319372 | Pit house | Schmitz et al. (2013b) |
| SC-CL-71 | 1270 ± 30 | Beta-319371 | Pit house | Schmitz et al. (2013b) |
| SC-CL-71 | 1260 ± 30 | Beta-329373 | Pit house | Schmitz et al. (2013b) |
| SC-CL-71 | 830 ± 30 | Beta-316467 | Pit house | Schmitz et al. (2013b) |
| SC-CL-71 | 370 ± 30 | Beta-316464 | Pit house | Schmitz et al. (2013b) |
| SC-CL-94 | 770 ± 40 | Beta-275576 | Mound and enclosure | Schmitz et al. (2013b) |
| SC-CR-06 | 220 ± 40 | Beta-190312 | Mound and enclosure | De Masi (2005) |
| SC-IÇ-01 | 1580 ± 60 | Beta-72196 | Surface site | Schmitz et al. (1999) |
| SC-IÇ-01 | 1470 ± 60 | Beta-72197 | Surface site | Schmitz et al. (1999) |
| SC-RA-01 | 1280 ± 40 | CAMS-51673 | Rock shelter | De Masi (2001) |
| SC-RA-03 | 1340 ± 40 | CAMS-54142 | Rock shelter | De Masi (2001) |
| SC-RA-05 | 1370 ± 40 | CAMS-51674 | Rock shelter | De Masi (2001) |
| SC-RA-06 | 1330 ± 40 | CAMS-54143 | Rock shelter | De Masi (2001) |
| SC-TA-04 | 1390 ± 50 | Beta-247953 | Pit house | Schmitz et al. (2009) |
| SC-TA-04 | 1220 ± 50 | Beta-228165 | Pit house | Schmitz et al. (2009) |
| SC-TA-04 | 1180 ± 40 | Beta-229856 | Pit house | Schmitz et al. (2009) |
| SC-TA-04 | 650 ± 50 | Beta-214107 | Pit house | Schmitz et al. (2009) |
| SC-U-35 | 975 ± 95 | SI-825 | Surface site | Schmitz and Brochado (1972) |
| SC-VI-16 | 1390 ± 50 | CAMS-53114 | Rock shelter | De Masi (2001) |
| Tapera | 1140 ± 180 | SI-245 | Surface site | Schmitz (1988) |
| Tapera | 1030 ± 180 | SI-246 | Surface site | Schmitz (1988) |
| Torre de Pedra | 270 ± 60 | N/A | Rock shelter | De Blasis (1996) |
| Urubici | 910 ± 200 | N/A | Rock shelter | Piazza (1966) |
| Urubici | 1840 ± 40 | CAMS-53915 | Rock shelter | De Masi (2001) |
| Walgimiro de Matos I | 730 ± 150 | N/A | Surface site | Parellada (2005) |

Table S3. Coefficient, standard deviation, odds ratio and significance for the terrain variables entered into the predictive model of forest distribution in the regions of Lages and Campo Belo do Sul. TPI= topographic position index.

|  | **Lages** |  |  |  |  | **Campo Belo do Sul** | |  |  |
| --- | --- | --- | --- | --- | --- | --- | --- | --- | --- |
|  | **β** | **s.d.** | **odds ratio** | **p** |  | **β** | **s.d.** | **odds ratio** | **p** |
| **Elevation** | 0.79538 | 0.19375 | 5.168125 | <0.01 |  | 0.385431 | 0.07487 | 2.598878 | <0.01 |
| **Slope** | 1.45765 | 0.17716 | 0.167152 | <0.01 |  | 0.038958 | 0.08549 | 1.126663 | 0.163 |
| **Aspect** | 0.85826 | 0.06083 | 1.85501 | <0.01 |  | 0.261069 | 0.03013 | 1.326906 | <0.01 |
| **TPI** | -1.33362 | 0.29666 | 0.016118 | <0.01 |  | 0.315595 | 0.10061 | 2.87505 | <0.01 |

Table S4. Table of isotopic results from soil organic matter from soil profiles in Campo Belo do Sul and Lages.

| Site | Location | Depth | Concentration C (%) | δ^13^C |
| --- | --- | --- | --- | --- |
| Mata Queimada | Lower south-facing | 0-5 | 7.86 | -28.5 |
| Mata Queimada | Lower south-facing | 05-10 | 2.71 | -23.81 |
| Mata Queimada | Lower south-facing | 10-15 | 2.14 | -21.58 |
| Mata Queimada | Lower south-facing | 15-20 | 4.93 | -20.29 |
| Mata Queimada | Lower south-facing | 20-25 | 2.11 | -17.96 |
| Mata Queimada | Lower south-facing | 25-30 | 1.79 | -15.97 |
| Mata Queimada | Lower south-facing | 30-35 | 1.78 | -17.19 |
| Mata Queimada | Lower south-facing | 35-40 | 1.63 | -16.13 |
| Mata Queimada | Lower south-facing | 40-45 | 1.56 | -18.01 |
| Mata Queimada | Lower south-facing | 45-50 | 1.34 | -16.8 |
| Mata Queimada | Lower south-facing | 50-55 | 1.27 | -17.72 |
| Mata Queimada | Lower south-facing | 55-60 | 1.09 | -20.43 |
| Mata Queimada | Upper south-facing | 0-5 | 4.02 | -27.32 |
| Mata Queimada | Upper south-facing | 05-10 | 2.74 | -24.27 |
| Mata Queimada | Upper south-facing | 10-15 | 2.07 | -22.62 |
| Mata Queimada | Upper south-facing | 15-20 | 2.05 | -22.2 |
| Mata Queimada | Upper south-facing | 20-25 | 1.75 | -21.49 |
| Mata Queimada | Upper south-facing | 25-30 | 1.62 | -20.18 |
| Mata Queimada | Plateau | 0-5 | 2.52 | -24.75 |
| Mata Queimada | Plateau | 05-10 | 2.04 | -24.15 |
| Mata Queimada | Plateau | 10-15 | 1.87 | -23.04 |
| Mata Queimada | Plateau | 15-20 | 2.2 | -22.65 |
| Mata Queimada | Plateau | 20-25 | 1.75 | -20.36 |
| Mata Queimada | Plateau | 25-30 | 1.69 | -19.91 |
| Mata Queimada | Plateau | 30-35 | 1.66 | -18.74 |
| Mata Queimada | Plateau | 35-40 | 1.46 | -18.47 |
| Mata Queimada | Plateau | 40-45 | 1.42 | -17.55 |
| Mata Queimada | Plateau | 45-50 | 1.38 | -17.56 |
| Mata Queimada | Plateau | 50-55 | 1.42 | -17.56 |
| Mata Queimada | Plateau | 55-60 | 1.47 | -18.25 |
| Mata Queimada | North-facing | 0-5 | 4.54 | -19.52 |
| Mata Queimada | North-facing | 05-10 | 3.2 | -20.84 |
| Mata Queimada | North-facing | 10-15 | 2.23 | -20.06 |
| Mata Queimada | North-facing | 15-20 | 1.77 | -18.84 |
| Heraldo | Plateau | 0-5 | 4.06 | -25.69 |
| Heraldo | Plateau | 05-10 | 2.78 | -23.94 |
| Heraldo | Plateau | 10-15 | 2.73 | -23.52 |
| Heraldo | Plateau | 15-20 | 2.2 | -21.19 |
| Heraldo | Plateau | 20-25 | 2.06 | -20 |
| Heraldo | Plateau | 25-30 | 1.89 | -19.41 |
| Heraldo | Plateau | 30-35 | 1.88 | -18.6 |
| Heraldo | Plateau | 35-40 | 1.83 | -17.73 |
| Heraldo | Plateau | 40-45 | 1.81 | -18.27 |
| Heraldo | Plateau | 45-50 | 1.59 | -17.74 |
| Heraldo | Plateau | 50-55 | 1.7 | -18.02 |
| Heraldo | Plateau | 55-60 | 1.7 | -18.29 |
| Heraldo | West-facing | 0-5 | No gás |  |
| Heraldo | West-facing | 05-10 | 5.74 | -23.24 |
| Heraldo | West-facing | 10-15 | 5.26 | -21.48 |
| Heraldo | West-facing | 15-20 | 4.96 | -20 |
| Heraldo | West-facing | 20-25 | 3.67 | -18.12 |
| Heraldo | West-facing | 25-30 | 2.87 | -17.34 |
| Heraldo | West-facing | 30-35 | 2.99 | -17.15 |
| Heraldo | West-facing | 35-40 | 2.79 | -17.81 |
| Heraldo | Riparian | 0-5 | 4.47 | -27.57 |
| Heraldo | Riparian | 05-10 | 3.29 | -25.68 |
| Heraldo | Riparian | 10-15 | 2.7 | -24.49 |
| Heraldo | Riparian | 15-20 | 2.09 | -22.22 |
| Heraldo | Riparian | 20-25 | 1.88 | -20.85 |
| Heraldo | Riparian | 25-30 | 1.79 | -19.71 |
| Heraldo | Riparian | 30-35 | 1.61 | -18.42 |
| Heraldo | Riparian | 35-40 | 1.5 | -17.64 |
| Heraldo | Riparian | 40-45 | 1.46 | -17.19 |
| Heraldo | Riparian | 45-50 | 1.36 | -17.06 |
| Heraldo | Riparian | 50-55 | 1.27 | -17.28 |
| Heraldo | Riparian | 55-60 | 1.4 | -18.55 |
| Luis Carlos | Plateau | 0-5 | 4.58 | -26.49 |
| Luis Carlos | Plateau | 05-10 | 2.99 | -23.21 |
| Luis Carlos | Plateau | 10-15 | 2.76 | -21.98 |
| Luis Carlos | Plateau | 15-20 | 2.41 | -21.14 |
| Luis Carlos | Plateau | 20-25 | 1.9 | -19.06 |
| Luis Carlos | Plateau | 25-30 | 1.73 | -19.35 |
| Luis Carlos | Plateau | 30-35 | 1.51 | -18.7 |
| Luis Carlos | Plateau | 35-40 | 1.29 | -18.12 |
| Luis Carlos | Plateau | 40-45 | 1.25 | -18.16 |
| Baggio | Plateau | 0-5 | 3.5 | -26.64 |
| Baggio | Plateau | 05-10 | 3.08 | -24.72 |
| Baggio | Plateau | 10-15 | 2.72 | -23.03 |
| Baggio | Plateau | 15-20 | 2.49 | -21.7 |
| Baggio | Plateau | 20-25 | 2.11 | -20.45 |
| Baggio | Plateau | 25-30 | 1.57 | -17.81 |
| Baggio | Plateau | 30-35 | 1.5 | -18.35 |
| Baggio | Plateau | 35-40 | 1.2 | -16.97 |
| Baggio | Plateau | 40-45 | 1.1 | -16.62 |
| Baggio | Plateau | 45-50 | 1.05 | -16 |
| Baggio | Plateau | 50-55 | 1.02 | -16.37 |
| Baggio | Plateau | 55-60 | 1.04 | -16.15 |
| Lages | North-facing | 0-5 | 3.12 | -16.47 |
| Lages | North-facing | 05-10 | 2.49 | -14.79 |
| Lages | North-facing | 10-15 | 2.19 | -14.09 |
| Lages | North-facing | 15-20 | 2.08 | -13.96 |
| Lages | North-facing | 20-25 | 1.88 | -14.1 |
| Lages | North-facing | 25-30 | 1.53 | -15.14 |
| Lages | North-facing | 30-35 | 1.37 | -16.14 |
| Lages | North-facing | 35-40 | 1.25 | -17.51 |
| Lages | North-facing | 40-45 | 1.17 | -16.71 |
| Lages | North-facing | 45-50 | 1.05 | -17.04 |
| Lages | North-facing | 50-55 | 1.04 | -18.5 |
| Lages | North-facing | 55-60 | 0.97 | -18.8 |
| Lages | Lower south-facing | 0-5 | 5.37 | -26.18 |
| Lages | Lower south-facing | 05-10 | 3.04 | -24.99 |
| Lages | Lower south-facing | 10-15 | 2.29 | -24.45 |
| Lages | Lower south-facing | 15-20 | 1.9 | -24.1 |
| Lages | Lower south-facing | 20-25 | 1.49 | -23.62 |
| Lages | Lower south-facing | 25-30 | 1.51 | -23.49 |
| Lages | Lower south-facing | 30-35 | 1.24 | -23.45 |
| Lages | Lower south-facing | 35-40 | 1.18 | -23.87 |
| Lages | Lower south-facing | 40-45 | 1.19 | -23.73 |
| Lages | Lower south-facing | 45-50 | 1.46 | -24.24 |
| Lages | Lower south-facing | 50-55 | 1.02 | -23.64 |
| Lages | Upper south-facing | 0-5 | 4.11 | -26.48 |
| Lages | Upper south-facing | 05-10 | 2.4 | -25.83 |
| Lages | Upper south-facing | 10-15 | 1.66 | -25.24 |
| Lages | Upper south-facing | 15-20 | 1.49 | -25 |
| Lages | Upper south-facing | 20-25 | 1.15 | -24.24 |
| Lages | Upper south-facing | 25-30 | 1.13 | -24.6 |
| Lages | Upper south-facing | 30-35 | 0.95 | -24.37 |
| Lages | Upper south-facing | 35-40 | 0.94 | -24.4 |
| Lages | Plateau | 0-5 | 8.63 | -15.74 |
| Lages | Plateau | 05-10 | 9.48 | -14.47 |
| Lages | Plateau | 10-15 | 8.02 | -13.65 |
| Lages | Plateau | 15-20 | 8.41 | -13.34 |
| Lages | Plateau | 20-25 | 5.21 | -12.56 |
| Lages | Plateau | 25-30 | 5.96 | -13.04 |
| Lages | Plateau | 30-35 | 5.21 | -13.24 |
| Lages | Plateau | 35-40 | 4.32 | -13.67 |
| Lages | Plateau | 40-45 | 3.08 | -13.24 |
| Lages | Plateau | 45-50 | 3.28 | -14.13 |
| Lages | Plateau | 50-55 | 2.93 | -14.29 |
| Lages | Plateau | 55-60 | 2.7 | -13.46 |

Table S5. AMS radiocarbon dates of soil organic matter from soil profiles in Campo Belo do Sul and Lages.

| Context | Depth (cm) | RCYBP | δ13C‰ | Laboratory number |
| --- | --- | --- | --- | --- |
| Baggio | 20-25 | 840 ± 30 | -19.3 | Beta-458500 |
| Baggio | 25-30 | 1420 ± 30 | -18.1 | Beta-458501 |
| Heraldo - West-facing | 15-20 | 260 ± 30 | -20.2 | Beta-477225 |
| Heraldo - West-facing | 20-25 | 410 ± 30 | -18.8 | Beta-477226 |
| Luis Carlos | 15-20 | 330 ± 30 | -21.3 | Beta-440912 |
| Luis Carlos | 20-25 | 970 ± 30 | -19.4 | Beta-440913 |
| Luis Carlos | 40-45 | 2350 ± 30 | -19.1 | Beta-440914 |
| Lages - Plateau | 55-60 | 6660 ± 30 | -14.1 | Beta-471037 |

**SI References**

Araujo AGDM (2001) *Teoria e método em arqueologia regional: um estudo de caso no alto Paranapanema, Estado de São Paulo*. PhD Dissertation, Universidade de São Paulo.

Brochado JJJP et al. (1969) Arqueologia Brasileira em 1968. *Publicações Avulsas do Museu Paraense Emilio Goeldi, 12*.

Bryan A (1965) Paleoamerican prehistory of Forte Marechal Luz. *Arquivos do Museu de Historia Natural da UFMG* 2: 9-30.

Chmyz I (1968) Considerações sobre duas novas tradições ceramistas arqueológicas no Estado do Paraná. *Pesquisas: Antropologia* 18: 115-125.

Chmyz I (1981) *Relatório das pesquisas arqueológicas realizadas na area da Usina Hidrelétrica de Salto Santiago (1979-80)*. Convênio Eletrosul-IPHAN, Curitiba.

Chmyz I (1995) Arqueologia de Curitiba. *Boletim Informativo da Casa Romário Martins* 21: 5-54.

Chmyz I, Bora E, Ceccon R, Sganzerla ME, Volcov JE (2003) A arqueologia da área do aterro Sanitário da região metropolitana de Curitiba, em Mandirituba, Paraná. *Revista do Centro de Ensino e Pesquisas Arqueológicas* 2: 1-138.

Chmyz I, Sganzerla E, Volcov JE, Bora E, Ceccon RS(2008) A arqueologia da área da LT 750 kV Ivaiporã - Itaberá III, Paraná - São Paulo. *Revista do Centro de Ensino e Pesquisas Arqueológicas* 5: 1-305.

Chmyz I, Sganzerla E, Volcov JE, Bora E, Ceccon RS (2009) *Relatório final do projeto de salvamento arqueológico na área de implantação da Mina Dois Irmãos, em São Mateus do Sul – Paraná*. Curitiba.

Copé SM (2006) *Les grands constructeurs précoloniaux du plateau du sud du Brésil: étude de paysages archéologiques à Bom Jesus, Rio Grande do Sul, Brésil*. PhD Dissertation, Universite de Paris I.

Corteletti R (2012) *Projeto arqueológico Alto Canoas - Paraca: um estudo da presença Jê no planalto Catarinense*. PhD Dissertation, Universidade de São Paulo.

De Blasis P (1996) *Bairro da Serra em três tempos: arqueologia, uso do espaço regional e continuidade cultural no médio vale do Ribeira.* PhD Dissertation, University of São Paulo.

De Blasis P, Farias DS, Kneip A (2014) Velhas tradições e gente nova no pedaço: perspectivas longevas de arquitetura funerária na paisagem do litoral sul catarinense. *Revista do Museu de Arqueologia e Etnologia* 24: 109-136.

De Masi MAN (2001) Pescadores coletores da costa Sul do Brasil. *Pesquisas: Antropologia* 57: 1-136.

De Masi MAN (2005) *Relatorio Final do Projeto de Salvamento Arqueologico UHE Campos Novos.* Unisul, Tubarao.

De Souza JG, Copé SM (2010) Novas perspectivas sobre a arquitetura ritual do planalto meridional brasileiro: pesquisas recentes em Pinhal da Serra, RS. *Revista de Arqueologia* 23: 98-111.

Farias DSE, Schmitz PI (2013) *Linguagem, dispersão e diversidade das populações Macro-Jê no Brasil meridional durante a pré-história brasileira. Palhoça*: Ed. Unisul.

Fossari TD (2004) A população pré-colonial Jê na paisagem da ilha de Santa Catarina. PhD Dissertation, UFSC.

Iriarte, J., Gillam, J. C. & Marozzi, O. 2008. Monumental burials and memorial feasting: an example from the southern Brazilian highlands. *Antiquity* 82: 947-961.
Iriarte J, Copé SM, Fradley M, Lockhart JJ, Gillam CJ (2013) Sacred landscapes of the southern Brazilian highlands: understanding southern proto-Jê mound and enclosure complexes. *J Anthropol Archaeol 32*: 74–96.

Miller ET (1969) Pesquisas arqueológicas efetuadas no noroeste do Rio Grande do Sul. *Publicações Avulsas do Museu Paraense Emilio Goeldi* 10: 33-54.

Müller LM (2007) *Casas subterâneas do planalto catarinense: estudo de caso do sítio SC-AG-107*. XIV Congresso da Sociedade de Arqueologia Brasileira (CD-ROM). Florianópolis. SAB.

Müller LM (2008) *Sobre índios e ossos: estudo de três sítios de estruturas anelares construídos para enterramento por populações que habitavam o vale do rio Pelotas no período pré-contato.* Master's Thesis, PUCRS.

Parellada CI (2005) *Estudo arqueológico no alto vale do rio Ribeira: área do gasoduto Bolívia-Brasil, trecho X, Paraná*. PhD Dissertation, Universidade de Sao Paulo.

Piazza WF (1966) *As Grutas de São Joaquim e Urubici*. UFSC, Florianópolis.

Ribeiro P (1974) Primeiras datações pelo método C14 para o vale do Caí, Rio Grande do Sul. *Revista do CEPA* 1: 16-22.

Ribeiro P (1980) Casas subterrâneas do Planalto meridional, município de Santa Cruz do Sul, Brasil. *Revista do CEPA* 9: 1-52.

Ribeiro P, Ribeiro CT (1985) Levantamentos arqueológicos no município de Esmeralda, RS, Brasil. *Revista do CEPA* 12: 49-105.

Rohr JA (1960) Pesquisas paleo-etnográficas na Ilha de Santa Catarina II. *Pesquisas: Antropologia 8*.

Saldanha JDM (2005) *Paisagem, lugares e cultura material: uma arqueologia espacial nas terras altas do sul do Brasil.* Master's Thesis, PUCRS.

Schmitz PI (1969) Algumas datas de carbono 14 de casas subterrâneas no planalto do Rio Grande do Sul. *Pesquisas: Antropologia* 20: 163-167.

Schmitz PI (1988). As tradições ceramistas do planalto sul-brasileiro. *Documentos* 2: 75-130.

Schmitz PI, Arnt FV, Beber MV, Rosa AO, Rogge JH (2009) Taió, no vale do rio Itajaí, SC: o encontro de antigos caçadores com as casas subterâneas. *Pesquisas: Antropologia* 67: 185-320.

Schmitz PI, Brochado J (1972) Datos para una secuencia cultural del Estado de Rio Grande do Sul, Brasil. *Gabinete de Arqueologia Publicações* 2: 1-20.

Schmitz PI, Rogge JH (2008) Um sítio da tradição cerâmica Aratu em Apucarana, PR. *Revista do Museu de Arqueologia e Etnologia* 18: 47-68.

Schmitz PI, Rogge JH, Novasco RV, Mergen NM, Ferrasso S (2013a) Boa Parada: um lugar de casas subterrâneas, aterros-plataforma e danceiro. *Pesquisas: Antropologia* 70: 133-195.

Schmitz PI, Rogge JH, Novasco RV, Mergen NM, Ferrasso S (2013b) Rincão dos Albinos: um grande sítio Jê meridional*. Pesquisas: Antropologia* 70: 65-131.

Schmitz PI et al. (2002) O projeto Vacaria: casas subterrâneas no planalto rio-grandense. *Pesquisas: Antropologia*: 11-105.

Schmitz PI et al. (1999) Içara: um jazigo mortuário no litoral de Santa Catarina. *Pesquisas: Antropologia* 55: 1-164.

Schwengber VL, Novasco RV, Mello AB (2012) Contribuições para a arqueologia do planalto catarinense: escavações no município de Passos Maia. *Cadernos do CEOM* 1: 169-189.

Wolf S, Machado NTG, Oliveira JLD (2016) Arqueologia regional entre o Forqueta e o Guaporé: O contexto de ocupação Jê pré-colonial no centro/nordeste do estado do Rio Grande do Sul. *Cadernos do LEPAARQ* 13: 173-196.
